# Convenient Synthesis of 3,4-Dichloro-5-hydroxy-2(5*H*)-Furanone Glycoconjugates

**DOI:** 10.3390/molecules16021011

**Published:** 2011-01-25

**Authors:** Edyta Gondela, Krzysztof Z. Walczak

**Affiliations:** Department of Organic Chemistry, Bioorganic Chemistry and Biotechnology, Silesian University of Technology, Krzywoustego 4, 44-100 Gliwice, Poland; E-Mail: edyta.gondela@polsl.pl (E.G.)

**Keywords:** 3,4-dichloromucochloric acid, amino alcohol, glucals, addition, glycoconjugate

## Abstract

3,4-Dichloro-5-hydroxy-2(5*H*)-furanone treated with methyl chloroformate in the presence of diisopropylethylamine (Hünig’s base) gave the corresponding carbonate. The labile methoxycarbonyloxy group smoothly undergoes substitution by amino alcohols. The obtained 5-(ω-hydroxyalkylamino) mucochloric acid derivatives reacted with peracetylated glucals using triphenylphosphine hydrobromide as a catalyst to give the title muchloric acid glycoconjugates.

## 1. Introduction

Mucochloric acid (3,4-dichloro-5-hydroxy-2(5*H*)-furanone, MCA, **1**) is a highly functionalized molecule possessing a hydroxyl group, two chlorine atoms and a lactone-like structure. A difference in reactivity of the two halogen atoms enables selective structural transformations [1]. Due to the presence of many reactive centers, MCA can be tailored into a useful building block for several synthetic purposes [2,3,4]. The 5-hydroxyl group present at C-5 position of the MCA molecule exhibits typical alcohol properties and enables its transformation into carbamates [5], ethers [5] and esters [5,6]. Chlorination of MCA can be performed using thionyl chloride in the presence of zinc chloride [6]. Esterification of the 5-hydroxyl group allows for its substitution by reactive nucleophiles like amines or alcohols. 5-Methoxycarbonyloxy-3,4-dichloro-2(5*H*)-furanone reacts with secondary amines at room temperature in toluene giving the corresponding 5-amino derivatives in yields of 61–79% [7]. The unsubstituted mucochloric acid can be coupled with aromatic thiols under acidic conditions furnishing 5-thiosubstituted derivatives in the 70–84% yield range [8]. MCA can be alkylated at the C-5 position in the aldol condensation catalyzed by Lewis acids [9]. 5-Allylation of MCA can be performed using allyl bromides in the presence of indium [10]. A treatment of MCA with ammonium formate or acetate in the presence of NaBH(OAc)_3_ leads to 3,4-dichloro-α,β-unsaturated butyrolactone [11]. *O*-Protected mucochloric acid reacted with appropriate amines giving 1,4-addition/elimination products [12,13]. The polarity of the reaction medium has a strong influence on the reaction course. In aprotic solvents, e.g. DMSO, the 4-substituted derivative of MCA is formed – formally, it is a product of addition/elimination reaction. In turn, in CHCl_3 _solution, a product of the ring opening and recyclisation is isolated [13]. Other nitrogen-centered nucleophiles can be involved in the reactions with MCA. For example, MCA treated with hydrazine or its derivatives forms the corresponding pyridazones [14,15]. Unsubstituted MCA condenses with racemic 2-amino-3-mercapto-3-methyl-butanoic acid forming a bicyclic β-lactam [16], whereas with formamides the products of substitution at carbon C4 of the 2-(5*H*)-furanone moiety are obtained [17]. Mucohalic acids also react with the amino group of purine nucleosides forming bicyclic systems or 3-amino-2-halopropenal derivatives [18,19].

Aliphatic or aromatic amines, amino acids and their esters may be successfully employed in a reductive amination of mucochloric acid [20,21]. The reaction is carried out at room temperature in the presence of NaBH(OAc)_3_ and a catalytic amount of AcOH in one of several solvents of choice. The best yields (52–75%) were achieved in the case of chloroform or dichloroethane as a solvent. 

The 2(5*H*)-furanone moiety occurs in numerous natural products exhibiting various biological activities [1,21,22,23,24]. The simple derivatives of mucochloric acid display interesting bioactivity. 5-Butoxy-3,4-dichloro-2(5*H*)-furanone is cytotoxic at 3 mM concentration against MAC 13 and MAC 16 murine colon cancer cell lines [5]. Further improvements of the leading structure furnished 3,4-dichloro-5-(oxiran-2-ylmethoxy)-2(5*H*)-furanone derivatives with cytotoxicity in the nanomolar range on the same cancer cell lines [5]. A broad antibiotic activity against *Staphylococcus aureus* ATCC 25923 and other bacteria strains has been observed in the case of 4-amino-5-hydroxy-2(5*H*)-furanones [17]. Recently, an inhibitory effect of 3,4-dichloro-5-(ω-hydroxyalkylamino)-2(5*H*)-furanones against human *Trypanosomas* has been published [25]. As a part of our investigation we report herein our initial trials of the synthesis of MCA conjugates. 

## 2. Results and Discussion

A conjunction of hydrophobic and hydrophilic moieties is a known strategy applied by Nature and copied on a large scale by the pharmaceutical industry to increase the bioavailability of drugs. The hydrophilic part can be represented by a sugar ring or an acyclic linker bearing hydroxyl or carboxylic groups. Valacyclovir, the L-valyl ester of acyclovir is a prodrug intended to increase the bioavailability of acyclovir by increasing lipophilicity [26]. In zalcitabine and lamivudine the cytosine is connected with a sugar-like ring [26]. The others are the derivatives of (*S*)-2-amino-3-(uracil-1-yl)propionic acid, the antagonists of kainate and AMPA receptors [27]. Therefore, the desired 3,4-dichloro-2(5*H*)-furanone conjugates should possess the furanone ring connected to the sugar moiety via an acyclic linker. 

**Scheme 1:**
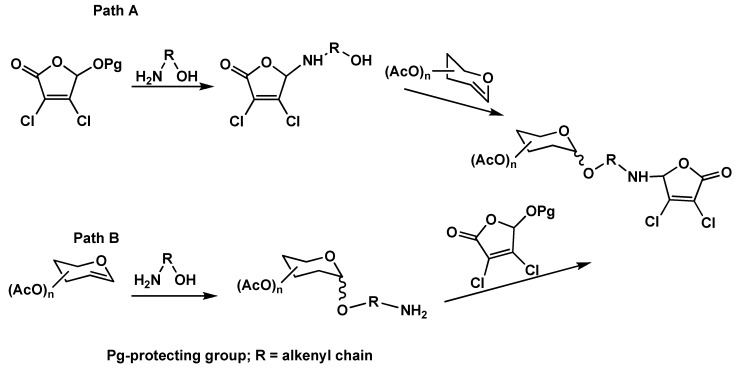
Possible synthetic pathways for the synthesis of MCA conjugates.

3,4-Dichloro-5-(ω-hydroxyalkylamino)-2(5*H*)-furanones were considered as starting materials having the ω-hydroxyalkylamino group at carbon C-5 of the 2(5*H*)-furanone ring suitable for the addition to the double bond of peracetylated glucals. There are two possible routes for this synthesis (Scheme 1). In path A, protected MCA is treated with an appropriate amino alcohol followed by the addition to peracetylated glucal in the presence of triphenylphosphine hydrobromide (TPHB) as a Lewis acid. It is known that the TPHB efficiently catalyzes the addition of alcohols to glucal derivatives without Ferrier rearrangement [28,29]. Path B differs in the reaction sequence: the amino alcohol is first added to glucal derivative forming an appropriate ω-aminoalkyl glycoside. In the next step, the final compounds can be obtained conveniently by the substitution of a good leaving group present at carbon C5 of 2(5*H*)-furanone, in most of the cases a protecting group.

**Scheme 2:**
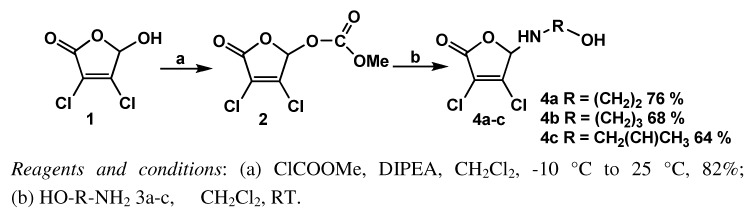
Synthesis of 3,4-dichloro-5-(ω-hydroxyalkylamino)-2(5*H*)-furanones.

In this paper we investigated the strategy outlined in the path A. In the first step, mucochloric acid (**1**) was transformed into its derivative containing a labile group at the carbon C-5. Thus, **1** was treated with methyl chloroformate in the presence of diisopropylethylamine and the appropriate carbonate **2** was obtained in 82% yield. Subsequently, the obtained derivative was subjected to the action of amino alcohols **3a**-**c** used as the *N*-centered nucleophiles in the substitution reaction. In a standard procedure, the mucochloric acid derivative **2** (1 equiv.) was treated with 2 equiv. of the amino alcohol in dichloromethane at room temperature (Scheme 2). TLC indicated total consumption of the limiting reactant after 0.5-2 h. The excess of amino alcohol was removed from the post-reaction mixture by evaporation under diminished pressure and the products were isolated using column chromatography. The yields of the products **4a**-**c** vary in the range of 64-76%. The disappearance of a characteristic signal from the carboxymethoxy group present at 3.93 ppm confirms the substitution at carbon C5 of 2(5*H*)-furanone ring.

**Scheme 3:**
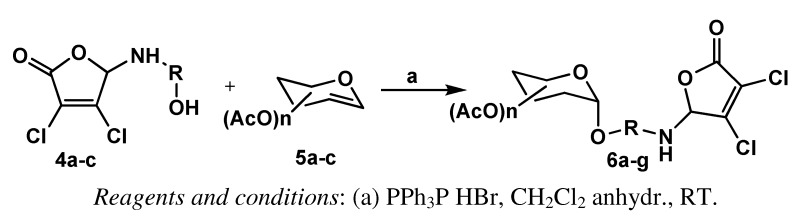
The coupling of MCA derivatives with peracetylated glucals.

**Table 1:** Addition of MCA derivatives to peracetylated glucals.

Entry	Conjugate	Peracetylated Glucal	MCA Derivative	Yield [%]
1	**6a**	D-Glucal	**4a**	98
2	**6b**		**4b**	78
3	**6c**		**4c**	78
4	**6d**	D-Galactal	**4a**	89
5	**6e**		**4b**	80
6	**6f**	L-Rhamnal	**4a**	78
7	**6g**		**4b**	60
8	**6h**		**4c**	61

The obtained 3,4-dichloro-5-(ω-hydroxyalkylamino)-2(5*H*)-furanones **4a**-**c** were used in the next step for the synthesis of conjugates (Scheme 3). As a sugar reactant we used commercially available peracetylated glucals. Preliminary trials carried out using 3,4,6-tri-*O*-acetyl-D-glucal **5a** and 5-(*N*-hydroxyethyl)amino-3,4-dichloro-2(5*H*)-furanone (**4a**) indicated that the addition reaction proceeded efficiently in anhydrous dichloromethane at room temperature when 1 equiv. of unsaturated sugar **5a** was treated with 1.1 equiv. of **4a**, in the presence of 0.2 equiv. of TPHB catalyst. The inspection of the reaction mixture after 24 h (TLC, AcOEt-hexane, 1:1, v/v) indicated formation of one product containing a sugar ring and a complete consumption of the substrate **4a**. The addition product **6a** was obtained in very good yield (Table 1, entry 1). The same ratio of reactants was applied in other experiments (Table 1, entry 2-8). The obtained yields of adducts **6b**-**h **are satisfying and vary in the range of 61-89%. 

The stereochemistry of the performed reaction is very complicated. The starting 3,4-dichloro-5-(ω-hydroxyalkylamino)-2(5*H*)-furanones **4a**-**c **constitute a racemic mixture bearing a chiral centre at carbon C5 of the furanone ring. Racemisation is a result of equilibrium between acyclic and cyclic forms of MCA in most solvents. The coupling with unsaturated sugars should lead theoretically to the formation of two possible anomers in respect to the anomeric carbon of sugar moiety. Inspection of ^1^H- and ^13^C-NMR spectra revealed the predominant diastereomer to be the α-D-anomer, and only traces of β-D-anomer were observed. In the ^1^H-NMR spectrum of compound **6a**, the anomeric proton appears as a doublet of doublets at 4.96 ppm, with coupling constants of 1.2 Hz and 3.6 Hz, suggesting an axial configuration for the aminoalkyl group/linker. The small value of *J* reflects the equatorial-equatorial relationship of the anomeric hydrogen with H-2_b_, the second 3.6 Hz one may be ascribed to H-2_a_–H-1 interaction. In all of the reactions involving MCA substrates **4a**–**c **and glucal **5a**, the main products possessed a *trans* configuration with respect to the acyl group present at carbon C-3 of the pyranosyl ring. For the L-rhamnose derivative, a β-L-configuration and *cis* in respect to the C-3 acyl group, was assigned. The observed stereoselectivity – in a respect to the anomeric centers – fortunately reduced the number of possible diastereomeric products **6** to two, apart from **6c** and **6h** where the linker had an additional stereogenic centre and another pair of diastereomers was created. Unfortunately, in this case the attempts to separate the diastereomers involving HPLC were unsatisfactory and failed.

## 3. Experimental

### 3.1. General

NMR spectra were recorded at 600 MHz for ^1^H-NMR and 150 MHz for ^13^C-NMR on a Varian 600 MHz System in CDCl_3_ or DMSO solutions; δ-values are in parts per million relative to tetramethylsilane used as an internal standard. Mass spectra were recorded at ESI Bruker-Daltonics Amazon Mass Spectrometer. TLC plates 60 F_254_ (Merck); visualized by UV light (254 nm) or using 3% solution of vanillin in MeOH containing 10% of H_2_SO_4_ and heating. Column chromatography was performed on silica gel packed column (silica gel 60; 0.040–0.063 mm, Merck) using solution of ethyl acetate/hexane v/v 1:2 or 1:1. Solvents were distilled prior to use and dried by standard methods. 5-Methoxycarbonyloxy-3,4-dichloro-2(5*H*)-furanone (**2**) and compounds **4a**-**c** were obtained according to reported procedures [7,25]. All reagents used were purchased from Lancaster. 

### 3.2. General procedure for the synthesis of 3,4-dichloro-5-( ω-hydroxyalkylamino)-2(5H)-furanones ***4***

To a mixture of 3,4-dichloro-5-methoxycarbonyloxy-2(5*H*)-furanone (1 equiv.) in anhydrous CH_2_Cl_2_ (5 mL), an appropriate amino alcohol 3a-c (2 equiv.) was slowly added while stirring. The reaction mixture was stirred at room temperature until the TLC (10% MeOH/CHCl_3_) revealed complete consumption of the limiting reactant (approx. 0.5-2 h). Then the solvent was evaporated under high vacuum and the residual oil was purified on a silica gel packed column using a mixture of MeOH and CHCl_3_ (1%, 3%, 5% or 10% v/v) as an eluent.

*3.2.1. 3,4-Dichloro-5-(2'-hydroxyethylamino)-2(5H)-furanone* (**4a**): White powder, m.p. 98-100 °C; ^1^H-NMR (DMSO-*d_6_*): δ 3.17-3.28 (m, 2H, H-1’), 3.49-3.58 (m, 2H, H-2’), 4.40 (br s, 1H, OH), 5.47 (s, 1H, H-5), 7.05 (s, 1H, NH). ^13^C-NMR (DMSO-*d_6_*): δ 42.20 (C-1'), 58.56 (C2'), 82.02 (C-5), 124.86 (C-3), 144.10 (C-4), 161.57 (C-2). Anal. Calcd. for C_6_H_7_Cl_2_NO_3_ (212): C, 33.99; H, 3.33; N, 6.60. Found: C, 33.87; H, 3.48; N, 6.41.

*3.2.2. 3,4-Dichloro-5-(3'-hydroxypropylamino)-2(5H)-furanone* (**4b**): White powder, m.p. 92-94 °C; ^1^H-NMR (DMSO-*d_6_*): δ 1.57-1.78 (m, 2H, H-2’), 3.26 (ddd, *J* = 6.1 Hz, *J* = 8.2 Hz, *J* = 14.1 Hz, 1H, H-1’a), 3.41 (t, *J* = 6.1 Hz, 2H, H-3’), 3.49 (m, 1H, H-1’b), 4.48 (s, 1H, OH), 5.43 (d, *J* = 9.3 Hz, 1H, H-5), 7.02 (d, *J* = 9.3 Hz, 1H, NH). ^13^C-NMR (DMSO-*d_6_*): δ 30.99 (C-2'), 37.48 (C-1'), 58.45 (C-3'), 81.62 (C-5), 124.75 (C-3), 143.94 (C-4), 161.43 (C-2). Anal. Calcd. for C_7_H_9_Cl_2_NO_3_ (226): C, 37.19; H, 4.01; N, 6.20. Found: C, 37.58; H, 4.01; N, 5.85.

*3.2.3. 3,4-Dichloro-5-(2'-hydroxypropylamino)-2(5H)-furanone* (**4c**): White powder, m.p. 86-88 °C; ^1^H NMR (DMSO-*d_6_*): δ 1.03 (d, *J* = 6.3 Hz, 3H, H-3'), 1.04 (d, *J* = 6.3 Hz, 3H, H-3'), 3.00-3.14 (m, 2 x 1H, H-1'a), 3.34-3.46 (m, 2 x 1H, H-1'b), 3.80-3.89 (m, 2 x 1H, H-2'), 4.83 (br s, 2 x 1H, OH), 5.45-5.51 (m, 2 x 1H, H-5), 7.00-7.05 (m, 2x1H, NH). ^13^C-NMR (DMSO-*d_6_*): δ 20.79, 21.15 (C-3'); 46.85, 47.04 (C1'), 63.99, 64.72 (C-2'), 81.88, 82.58 (C-5), 124.84, 124.87 (C-3); 143.95, 144.21 (C-4), 161.54, 161.81 (C-2). Anal. Calcd. for C_7_H_9_Cl_2_NO_3_ (226): C, 37.19; H, 4.01; N, 6.20. Found: C, 36.86; H, 3.67; N, 6.17.

### 3.2. General procedure for the synthesis of compounds ***6a-g***

The appropriate 5-(*N*-hydroxyalkyl)amino-3,4-dichloro-2(5*H*)-furanone **4a-c** (1.1 equiv., 0.1 g) was dissolved in anhydrous CH_2_Cl_2_ (6 mL) and then 1,2-unsaturated sugar **5a-c** (1 equiv.) and TPHB (0.2 equiv.) were added. The reaction mixture was stirred for 24 h at room temperature (TLC, AcOEt : Hexane, 1:1_,_ v/v). Then the solvent was evaporated under reduced pressure, the residue was purified using silica gel packed column and a mixture of AcOEt and hexane (1:2, 1:1, v/v) as an eluent. The combined fraction-containing products **6a g **were collected and evaporated under diminished pressure. The products were obtained in satisfactory yield as semi-solid materials.

*2-(3,4-Dichloro-5-oxo-2,5-dihydrofuran-2-ylamino)ethyl 3,4,6-tri-O-acetyl-2-deoxy α-D arabinohexo- pyranoside* (**6a**): ^1^H-NMR (CDCl_3_) δ (ppm): 1.83 (ddd, 1H, *J* = 3.6 Hz, 11.7 Hz, 13.1 Hz, H-2_a_), 2.02 (s, 3H, CH_3_), 2.05 (s, 3H, CH_3_), 2.10 (s, 3H, CH_3_), 2.25 (ddd, 1H, *J* = 1.2 Hz, 5.6 Hz, 13.1 Hz, H-2_b_), 3.59-3.66 (m, 2H, H-1’), 3.82-3.88 (m, 2H, H-2’), 4.04 (dd, 1H, *J* = 3.0 Hz, 4.8 Hz, H-5), 4.21 (dd, 1H, *J* = 4.8 Hz, 12.3 Hz, H-6_b_), 4.25 (dd, 1H, *J* = 4.8 Hz, 12.3 Hz, H-6_a_), 4.96 (dd, 1H, *J* = 1.2 Hz, 3.6 Hz, H-1), 5.0 (dd, 1H *J* = 3.0 Hz, 9.5 Hz, H-4), 5.19 (ddd, 1H, *J* = 5.6 Hz, 9.5 Hz, 11.7 Hz, H-3), 5.42 (d, 1H, *J* = 2.7 Hz, H-5’’), NH group not observed. ^13^C-NMR (CDCl_3_) δ (ppm): 20.75, 20.80, 20.98 (3×CH_3_), 34.82, 41.45 (C-2, C-1’), 62.45, 65.51 (C-6, C-2’), 68.39, 68.99, 69.07 (C-3, C-4, C-5), 83.10 (C-5’’), 96.78 (C-1), 126.50 (C-3’’), 143.73 (C-4’’), 162.84 (C-2’’), 169.82, 170.73, 170.83 (3×C=O). ESI-MS: *m/z* 484.0 ([M+H]^+^, C_18_H_23_Cl_2_NO_10_H^+^, calcd. 484.1).

*3-(3,4-Dichloro-5-oxo-2,5-dihydrofuran-2-ylamino)propyl 3,4,6-tri-O-acetyl-2-deoxy α-D arabino-hexo-pyranoside* (**6b)**: ^1^H-NMR (CDCl_3_) δ (ppm): 1.81 (ddd, 1H, *J* = 3.0 Hz, 12.0 Hz, 13.2 Hz, H-2_a_), 1.90-1.99 (m, 2H, H-2’), 2.02 (s, 3H, CH_3_), 2.05 (s, 3H, CH_3_), 2.09 (s, 3H, CH_3_), 2.21 (ddd, 1H, *J* = 1.2 Hz, 4.8 Hz, 13.2 Hz, H-2_b_), 3.42-3.46 (m, 1H, H-3’_a_), 3.55-3.63 (m, 3H, H-5, H-1’) 3.71-3.74 (m, 1H, H-3’_b_), 4.05 (dd, 1H, *J* = 2.5 Hz, 12.2 Hz, H-6_b_), 4.27 (dd, 1H, *J*= 4.8 Hz, 12.2 Hz, H-6_a_), 4.92 (d, 1H, *J* = 3.0 Hz, H-1), 4.97 (dd, 1H, *J* = 5.4 Hz, 10.2 Hz, H-4), 5.19-5.24 (m, 1H, H-3), 5.34 (d, 1H, *J* = 5.1 Hz, H-5’’), NH group not observed. ^13^C-NMR (CDCl_3_) δ (ppm): 20.86 (2×C), 21.10 (3×CH_3_), 28.29 (C-2’), 34.98, 38.26 (C-2, C-1’), 62.63, 65.65 (C-6, C-3’), 68.03, 69.30, 69.42 (C-3, C-4, C-5), 82.81 (C-5’’), 97.29 (C-1), 126.62 (C-3’’), 143.52 (C-4’’), 163.01 (C-2’’), 170.06, 170.75, 171.05 (3×C=O). ESI-MS: *m/z* 498.1 ([M+H]^+^, C_19_H_25_Cl_2_NO_10_H^+^, calcd. 498.1).

*1-(3,4-Dichloro-5-oxo-2,5-dihydrofuran-2-ylamino)propan-2-yl 3,4,6-tri-O-acetyl-2 deoxy-α-D arab- inohexopyranoside* (**6c**): ^1^H-NMR (CDCl_3_) δ (ppm): 0.93 (d, 3H, *J* = 6.0 Hz, CH*CH_3_*), 1.79-1.86 (m, 1H, H-2_a_), 2.02 (s, 3H, CH_3_), 2.06 (s, 3H, CH_3_), 2.10 (s, 3H, CH_3_), 2.18-2.24 (m, 1H, H-2_b_), 3.55-3.63 (m, 3H, OC*H*, H-2’), 3.77-4.13 (m, 2H, H-5, H-6_b_), 4.27 (dd, 1H, *J* = 4.8 Hz, *J* = 12.3 Hz, H-6_a_), 4.93-5.03 (m, 2H, H-1, H-4), 5.17 (ddd, 1H, *J* = 5.2 Hz, *J* = 9.4 Hz, 11.5 Hz, H-3), 5.31 (d, 1H, *J* = 8.4 Hz, H-5’’), NH group not observed. ^13^C-NMR (CDCl_3_) δ (ppm): 20.76 (2×C), 20.97, 21.61 ((3×CH_3_, C-1’), 34.60 (C-2), 55.94, 62.37, 67.27 (C-6, C-2’, C-3’), 68.12, 69.03, 69.35 (C-3, C-4, C-5), 83.26 (C-5’’), 97.52 (C-1), 125.92 (C-3’’), 143.54 (C-4’’), 163.82 (C-2’’), 169.95, 170.34, 170.82 (3×C=O). ESI-MS: *m/z* 498.0 ([M+H]^+^, C_19_H_25_Cl_2_NO_10_H^+^, calcd. 498.1).

*2-(3,4-Dichloro-5-oxo-2,5-dihydrofuran-2-ylamino)ethyl 3,4,6-tri-O-acetyl-2-deoxy-α-D lyxohexo- pyranoside* (**6d**): ^1^H-NMR (CDCl_3_) δ (ppm): 1.87 (dd, 1H, *J* = 3.0 Hz, 12.6 Hz, H-2_a_), 2.00 (s, 3H, CH_3_), 2.07 (s, 3H, CH_3_), 2.08-2.13 (m, 1H, H-2_b_), 2.14 (s, 3H, CH_3_), 3.58-3.66 (m, 2H, H-1’), 3.77-3.86 (m, 2H, H-2’), 4.05 (dd, 1H, *J* = 1.8 Hz, 13.5 Hz, H-6_b_), 4.09 (dd, 1H, *J* = 1.8 Hz, 6.6 Hz, H-5), 4.12 (dd, 1H, *J* = 6.6 Hz, 13.5 Hz, H-6_a_), 5.05 (d, 1H, *J* = 4.8 Hz, H-4), 5.18 (dd, 1H, *J* = 3.0 Hz, 4.8 Hz, H-3), 5.23 (d, 1H, *J* = 3.0 Hz, H-1), 5.45 (d, 1H, *J* = 7.9 Hz, H-5’’), NH group not observed. ^13^C- NMR (CDCl_3_) δ (ppm): 20.71, 20.76, 20.92 (3×CH_3_), 30.03, 41.00 (C-2, C-1’), 62.46, 65.71 (C-6, C-2’), 66.19, 66.46, 67.14 (C-3, C-4, C-5), 83.10 (C-5’’), 97.48 (C-1), 126.59 (C-3’’), 143.63 (C-4’’), 162.86 (C-2’’), 170.24, 170.78, 170.87 (3×C=O). ESI-MS: *m/z* 484.0 ([M+H]^+^, C_18_H_23_Cl_2_NO_10_H^+^, calcd. 484.1).

*3-(3,4-Dichloro-5-oxo-2,5-dihydrofuran-2-ylamino)propyl 3,4,6-tri-O-acetyl-2-deoxy α-D lyxohexo- pyranoside* (**6e**): ^1^H-NMR (CDCl_3_) δ (ppm): 1.83 (dd, 1H, *J* = 3.6 Hz, 12.6 Hz, H-2_a_), 1.91-1.96 (m, 2H, H-2’), 1.99 (s, 3H, CH_3_), 2.06 (s, 3H, CH_3_), 2.07-2.11 (m, 1H, H-2_b_), 2.14 (s, 3H, CH_3_), 3.45-3.49 (m, 1H, H-3’_a_), 3.52-3.66 (m, 2H, H-1’), 3.70-3.75 (m, 1H, H-3’_b_), 4.07 (ddd, 1H, *J* = 1.8 Hz, 4.8 Hz, 6.0 Hz, H-5), 4.09-4.19 (m, 2H, H-6), 4.95 (t, 1H, *J* = 4.8 Hz, H-4), 5.22 (dd, 1H, *J* =1.8 Hz, 4.8 Hz, H-3), 5.28 (d, 1H, *J* = 3.6 Hz, H-1), 5.33 (d, 1H, *J* = 7.2 Hz, , H-5’’), NH group not observed. ^13^C- NMR (CDCl_3_) δ (ppm): 20.73, 20.90, 21.06 (3×CH_3_), 28.47 (C-2’), 30.11, 38.55 (C-2, C-1’), 60.46, 62.51 (C-6, C-3’), 65.47, 66.30, 66.53 (C-3, C-4, C-5), 82.75 (C-5’’), 97.66 (C-1), 126.68 (C-3’’), 143.28 (C-4’’), 162.92 (C-2’’), 170.36, 170.90, 171.19 (3×C=O). ESI-MS: *m/z* 498.0 ([M+H]^+^, C_19_H_25_Cl_2_NO_10_H^+^, calcd. 498.1).

*2-(3,4-Dichloro-5-oxo-2,5-dihydrofuran-2-ylamino)ethyl 3,4-di-O-acetyl-2,6-dideoxy-β L arabino-hexopyranoside* (**6f**): ^1^H NMR (CDCl_3_) δ (ppm): 1.17 (d, 3H, *J* = 7.2 Hz, H-6), 1.77-1.83 (dt, 1H, *J* = 3.0 Hz, 10.5 Hz, 12.6 Hz, H-2_a_), 2.02 (s, 3H, CH_3_), 2.05 (s, 3H, CH_3_), 2.21 (ddd, 1H, J = 1.8 Hz, 4.8 Hz, 12.6 Hz, H-2_b_), 3.57-3.64 (m, 2H, H-1’), 3.78-3.87 (m, 2H, H-2’), 4.12 (q, 1H, *J* = 7.2 Hz, H -5), 4.73 (dd, 1H, *J* = 4.8 Hz, 7.2 Hz, H-4), 4.92 (d, 1H, *J* = 3.0 Hz, H-1) 5.18 (ddd, 1H, *J* = 1.8 Hz, 4.8 Hz, 10.5 Hz, H-3), 5.43 (d, 1H, *J* = 7.1 Hz, H-5’’), NH group not observed. ^13^C-NMR (CDCl_3_) δ (ppm): 17.54 (C-6), 20.84, 21.04 (2×CH_3_), 35.11, 40.88 (C-2, C-1’), 65.48, 66.36, 68.99, 74.33 (C-2’, C-3, C-4, C-5), 83.38 (C-5’’), 96.75 (C-1), 126.48 (C-3’’), 143.77 (C-4’’), 162.83 (C-2’’), 170.08, 170.69 (2×C=O). ESI-MS: *m/z* 426.0 ([M+H]^+^, C_19_H_25_Cl_2_NO_10_H^+^, calcd. 426.1).

*3-(3,4-Dichloro-5-oxo-2,5-dihydrofuran-2-ylamino)propyl 3,4-di-O-acetyl-2,6-dideoxy-β L arabino-hexopyranoside* (**6g)**: ^1^H-NMR (CDCl_3_) δ (ppm): 1.17 (d, 3H, *J* = 6.6 Hz, H-6), 1.79 (ddd, 1H, *J* = 1.8 Hz, 4.8 Hz, 12.6 Hz, H-2_a_), 1.90-1.97 (m, 2H, H-2’), 2.01 (s, 3H, CH_3_), 2.06 (s, 3H, CH_3_), 2.21 (ddd, 1H, *J* = 1.2 Hz, 5.4 Hz, 12.6 Hz, H-2_b_), 3.43 (ddd, 1H, *J* = 4.5 Hz, 8.0 Hz, 11.3 Hz, H-3’_a_), 3.68-3.75 (m, 2H, H-1’), 3.77-3.84 (m, 1H, H-3’_b_), 4.11 (dd, 1H, *J* = 6.6 Hz, 9.6 Hz, H-5), 4.73 (dd, 1H, *J* = 1.8 Hz, 9.6 Hz, H-4), 4.84 (dd, 1H, *J* = 1.2 Hz, 4.8 Hz, H-1), 5.13 (ddd, 1H, *J* = 1.8 Hz, 5.4 Hz, 9.6 Hz, H-3), 5.35 (d, 1H, *J* = 6.1 Hz, H-5’’), NH group not observed. ^13^C-NMR (CDCl_3_) δ (ppm): 17.55 (C-6), 20.86, 21.04 (2×CH_3_), 28.32 (C-2’), 35.18, 38.51 (C-2, C-1’), 65.39, 65.98, 69.22, 74.51 (C-3’, C-3, C-4, C-5), 82.68 (C-5’’), 96.87 (C-1), 126.62 (C-3’’), 143.40 (C-4’’), 162.91 (C-2’’), 170.19, 170.74 (2×C=O). ESI-MS: *m/z* 462.1 ([M+Na]^+^, C_19_H_25_Cl_2_NO_10_Na^+^, calcd. 462.1).

*1-(3,4-Dichloro-5-oxo-2,5-dihydrofuran-2-ylamino)propan-2-yl 3,4-di-O-acetyl- 2,6 dideoxy β-L arabino-hexopyranoside* (**6h**): Mixture of diastereoisomers: ^1^H-NMR (CDCl_3_) δ (ppm): 1.10-1.30 (m, 6H, H-6, H-1’), 1.76-1.82 (m, 1H, H-2_a_), 2.03 (s, 3H, CH_3_), 2.06 (s, 3H, CH_3_), 2.08-2.16 (m, 1H, H-2_b_), 3.56-4.20 (m, 4H, H-5, H-2’, H-3’), 4.68-4.73 (m, 1H, H-4), 5.03-5.05 (m, 1H, H-1), 5.11-5.18 (m, 1H, H-3), 5.49 (d, 1H, *J* = 7.1 Hz, H-5’’), NH group not observed. ^13^C-NMR (CDCl_3_) δ (ppm): 17.36, 18.95 (C-6, C-1’), 20.82, 21.09 (2×CH_3_), 35.90 (C-2), 45.73, 66.72, 69.02, 69.40, 74.12 (C-3, C-4, C-5, C-2’, C-3’), 83.27 (C-5’’), 97.35 (C-1), 126.42 (C-3’’), 143.86 (C-4’’), 163.15 (C-2’’), 170.07, 170.97 (2×C=O). ESI-MS: *m/z* 462.1 ([M+Na]^+^, C_19_H_25_Cl_2_NO_10_Na^+^, calcd. 462.1).

## 4. Conclusions

We have synthesized a novel group of 3,4-dichloro-5-hydroxy-2(5*H*)-furanone glycoconjugates by the reaction of 3,4-dichloro-5-(ω-hydroxyalkylamino)-2(5*H*)-furanones with appropriate unsaturated sugars using TPHB as the condensing agent. The formation of conjugates proceeded stereospecifically in respect to the sugar ring. The final compounds will be tested for antiprotozoal activity within the framework of the Drugs for Neglected Diseases initiative (DND*i*).
